# Repeated Assessment of Exploration and Novelty Seeking in the Human Behavioral Pattern Monitor in Bipolar Disorder Patients and Healthy Individuals

**DOI:** 10.1371/journal.pone.0024185

**Published:** 2011-08-30

**Authors:** Arpi Minassian, Brook L. Henry, Jared W. Young, Virginia Masten, Mark A. Geyer, William Perry

**Affiliations:** Department of Psychiatry, University of California San Diego, San Diego, California, United States of America; The University of Queensland, Australia

## Abstract

**Background:**

Exploration and novelty seeking are cross-species adaptive behaviors that are dysregulated in bipolar disorder (BD) and are critical features of the illness. While these behaviors have been extensively quantified in animals, multivariate human paradigms of exploration are lacking. The human Behavioral Pattern Monitor (hBPM), a human version of the animal open field, identified a signature pattern of hyper-exploration in manic BD patients, but whether exploratory behavior changes with treatment is unknown. The objective of this study was to assess the sensitivity of the hBPM to changes in manic symptoms, a necessary step towards elucidating the neurobiology underlying BD.

**Methodology and Principal Findings:**

Twelve acutely hospitalized manic BD subjects and 21 healthy volunteers were tested in the hBPM over three sessions; all subjects were retested one week after their first session and two weeks after their second session. Motor activity, spatial and entropic (degree of unpredictability) patterns of exploration, and interactions with novel objects were quantified. Manic BD patients demonstrated greater motor activity, extensive and more unpredictable patterns of exploration, and more object interactions than healthy volunteers during all three sessions. Exploration and novelty-seeking slightly decreased in manic BD subjects over the three sessions as their symptoms responded to treatment, but never to the level of healthy volunteers. Among healthy volunteers, exploration did not significantly decrease over time, and hBPM measures were highly correlated between sessions.

**Conclusions/Significance:**

Manic BD patients showed a modest reduction in symptoms yet still demonstrated hyper-exploration and novelty seeking in the hBPM, suggesting that these illness features may be enduring characteristics of BD. Furthermore, behavior in the hBPM is not subject to marked habituation effects. The hBPM can be reliably used in a repeated-measures design to characterize exploration and novelty seeking and, in parallel with animal studies, can contribute to developing treatments that target neuropsychiatric disease.

## Introduction

Exploration is the act of making the unknown known and is a fundamental adaptive behavior across many species. A related adaptive behavior is novelty seeking, defined as a proclivity to approach unfamiliar situations. Abnormal exploratory behavior and novelty seeking are characteristic of many neuropsychiatric conditions, including excessive activity observed in bipolar mania, increased novelty seeking in substance use disorders, and prominent inactivity and withdrawal as observed in schizophrenia. For several decades, numerous animal paradigms of neuropsychiatric illness have assessed the multiple dimensions of exploratory behavior and novelty seeking [1], [2]. These models have been useful in elucidating underlying neurobiological mechanisms and testing novel psychotropic treatments. While some efforts have been made towards developing and validating quantitative human exploratory paradigms [3], [4], most of the work in this arena has focused on single measures of activity, such as actigraphy in children with Bipolar Disorder (BD) or attention deficit hyperactivity disorder (ADHD) [4], [5], and interaction with novel stimuli in autism [6].

In an effort to create a human paradigm that enables a multivariate assessment of exploration, we developed the human Behavioral Pattern Monitor (hBPM) [7], [8], the human analog of our rodent BPM [9], which is an elaboration of the traditional animal open field test. We have established that the hBPM yields distinct patterns of exploratory behavior in individuals in the manic phase of BD as compared to schizophrenia patients and non-patients in three fundamental dimensions of exploration [1], [2]: amount of motor activity [7], [10]; the sequential structure of this activity [7]; and the exploration of novel stimuli [11]. Specifically, manic BD patients demonstrated high levels of motor activity in the hBPM characterized by long, straight movements, as well as increased exploration of novel objects. Similar to the human findings, mice with reduced expression of the dopamine transporter (DAT), a putative model of BD, exhibit a strikingly comparable pattern of activity in the rodent BPM characterized by elevated motor activity, increased exploration, and patterns of straight movements [7]. What is unknown is whether this signature pattern of hyper-exploration persists in non-manic phases of BD. Of note, some literature suggests that increased novelty seeking does not appear to be limited to the manic state and has been observed in stable BD patients [12], but such studies have been limited to the use of self-report measures. While exploration and novelty seeking represent crucial phenotypes of BD, objective assessment of these constructs across different states of the illness is lacking.

Although the hBPM has shown validity in characterizing the manic state, the reliability of this measure over time and the potential sensitivity to alterations in mania symptoms has not been addressed. Furthermore, the extent of habituation to the hBPM environment has not been assessed in either healthy non-patients or less symptomatic BD patients. To characterize the relationship between exploratory behavior and changes in manic symptoms and further validate the hBPM as a useful measure of human exploration and novelty seeking, we quantified the behavior of a cohort of manic BD patients and healthy volunteers over the course of three sessions in the hBPM. We hypothesized that BD individuals would reliably show heightened motor activity and novel object exploration compared to healthy volunteers over the course of repeated testing, despite potential improvement in manic symptoms. We further hypothesized that healthy volunteers would show consistent levels of activity and exploration across the three sessions.

## Methods

### Subjects

This study was approved by the UCSD Institutional Review Board, known as the Human Research Protections Program (HRPP). Written informed consent was obtained from all subjects. Acutely hospitalized inpatients with SCID (Structured Clinical Interview for DSM-IV) diagnosed DSM-IV Bipolar Disorder, Current Episode Manic (n = 12, 6 M), between the ages of 18 to 55 were recruited for the study. Non-patient comparison (NC) participants who had never met criteria for an Axis I Disorder as determined by the SCID were recruited from the community (n = 21, 11 M). Participants were excluded for alcohol or substance abuse or dependence within the past month, a positive result on a urine toxicology screen, any neurological conditions, or medical conditions that impaired motor functioning.

### Procedure

Manic BD patients were tested in the hBPM within 96 hours of being admitted to an inpatient psychiatric unit (Session 1). The findings from Session 1 have been reported elsewhere [7], [10], [11]; the current report includes data from a second hBPM test session one week after Session 1 (Session 2), and a third test session two weeks after Session 2 (Session 3). NC subjects were also re-tested one week after Session 1, and again two weeks after Session 2. The Young Mania Rating Scale (YMRS) [13] was administered to BD subjects at all three sessions. As shown in Table 1, the majority of BD patients were taking an atypical antipsychotic agent and a mood-stabilizing medication, most commonly risperidone and valproate, respectively.

**Table 1:** Daily doses of antipsychotic and mood stabilizer medications for BD-manic patients (n  = 12).

Subject number	Session 1	Session 2	Session 3
1	None	valproate 1000 mg	valproate 2000 mg
		risperidone 1 mg	risperidone 4 mg
2	lamotrigine 150 mg	lamotrogine 75 mg	lamotrigine 125 mg
	oxcarbazepine 200 mg	oxcarbazepine 200 mg	oxcarbazepine 200 mg
3	valproate 2500 mg	valproate 1750 mg	valproate 2500 mg
	quetiapine 200 mg	olanzapine 5 mg	olanzapine 30 mg
	olanzapine 15 mg		
4	valproate 1500 mg	valproate 1500 mg	valproate 1500 mg
	mgrisperidone 2 mg	mgrisperidone 4 mg	mgrisperidone 4 mg
	mgziprasidone 80 mg		
5	lithium 300 mg	lithium 900 mg	lithium 900 mg
	risperidone 1 mg	risperidone 4 mg	risperidone 4 mg
	olanzapine 10 mg		
6	olanzapine 10 mg	none	none
7	lithiuim 900 mg	valproate 2000 mg	risperidone 2 mg
	risperidone 1 mg	risperidone 2 mg	
8	valproate 2000 mg	valproate 2000 mg	oxcarbazepine 900 mg
	risperidone 2 mg		risperidone 6 mg
	aripiprazole 15 mg		aripiprazole 30 mg
9	olanzapine 10 mg	valproate 2500 mg	valproate 2500 mg
	aripiprazole 15 mg	risperidone 8 mg	risperidone 8 mg
10	risperidone 2 mg	oxcarbazepine 5 mg	valproate 5000 mg
		risperidone 2 mg	oxcarbazepine 600 mg
			risperidone 2 mg
			olanzapine 15 mg
11	carbamazepine 400 mg	carbamazepine 400 mg	carbamazepine 400 mg
	aripiprazole 30 mg	aripiprazole 30 mg	aripiprazole 30 mg
12	valproate 2500 mg	valproate 2500 mg	valproate 2500 mg
	risperidone 6 mg	risperidone 6 mg	risperidone 6 mg

For the hBPM testing, subjects were fitted with an ambulatory monitoring device in the form of a wearable upper-body garment that resembles a sleeveless vest [14]. They were then directed into the hBPM and told that they would see the experimenter in a short period of time and to wait in the room. No other instructions were provided.

The hBPM is a 2.7 m×4.3 m room to which the subject has not been exposed previously. It contains several items of furniture placed along the periphery of the room, including a desk, small and large filing cabinets, and two sets of bookshelves. There is no chair in the room. Dispersed evenly on items of furniture are 11 small objects. These objects were chosen using the criteria that they be safe, colorful, tactile, manipulable, and likely to invite human exploration [6]. Subjects were left in the hBPM for 15 minutes. Their activity was monitored by a digital video camera embedded in the ceiling. Although all three hBPM sessions included 11 objects, these objects were changed for each session in an effort to maximize the novelty of the environment. For example, a mask in Session 1 was replaced by a hat in Session 2 and a pair of glasses in Session 3, thus changing the actual object but retaining its vital characteristic (e.g., something that can be worn).

The digitized video images were stored at 30 frames per second and subjected to frame-by-frame analysis with proprietary software [15] that generates x-y-coordinates of the subject's successive locations. The x-y coordinate data provide a measure of the amount of motor activity, quantified by counts, which are defined as the number of discrete instances of movement or the smallest measured change in x-y coordinates. Higher counts suggest more motor activity. Movement sequence and structure was also quantified from the x-y coordinates using the spatial scaling exponent *d* and dynamical entropy *h*. Spatial *d*, a measure of the hierarchical and geometric organization of behavior, is based on the principles of fractal geometry and describes the degree to which the path taken within an enclosure by the subject is one-dimensional or two-dimensional. To obtain spatial *d*, the measured distance traveled is plotted against different ruler lengths (used to quantify distance with varying degrees of precision) using a double-logarithmic coordinate system, and a line of fit between these two variables is generated [9], [16], [17]. Spatial *d* typically varies between 1 (a straight line) and 2 (a filled plane), with values closer to 1 reflecting straight movements and values closer to 2 reflecting highly circumscribed, local movements. At both ends of this spectrum, the geometric pattern of movement around the hBPM is highly predictable but exhibits either an almost straight-line movement or a highly circumscribed geometrical pattern, respectively [18]. Intermediate spatial *d* values, e.g., 1.5, suggest a mix of straight movements as well as localized and circumscribed movements. Dynamical entropy *h* measures the degree to which behavior is observed along a continuum between complete order and disorder [16]. To calculate *h*, a given sequence of movements is compared to similar preceding sequences, and this comparison is conducted for varying sequence lengths. Lower values of *h* (low entropy) suggest highly predictable or ordered patterns of movement across time, while higher values (high entropy) suggest a greater variety, or disorder, in the structure of movement across time.

In addition, the digitized video images enabled detailed assessments of the subject's interactions with the 11 objects placed in the room. The number of discrete interactions with the objects was hand-counted and recorded by experimenters who were blind to group membership. Video raters received at least one week of training with definitions and practice coding of videotapes of both patient and non-patient participants. Reliability checks were conducted over the course of the study, with kappa reliability coefficients for rater-coded measures ranging from 0.91 to 0.96.

**Figure 1:**
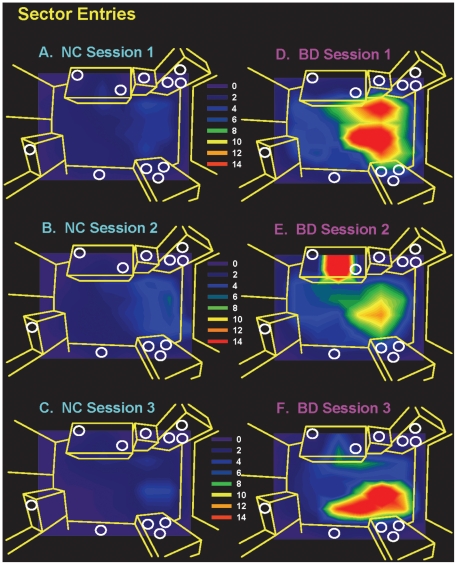
Contour maps of hBPM entries for manic BD and NC subjects. Circles depict object locations and brighter colors depict relatively more entries. In all three sessions, manic BD subjects showed more entries into areas of the hBPM that contain multiple objects.

Finally, a grid of 64 sectors of equal sizes was generated for the hBPM. The x-y coordinate data were derived from digitized videos of the hBPM session as previously described [19], and number of entries into each sector was quantified. Figure 1 displays the contour maps illustrating the distribution of average sector entries for NC and BD subjects for all three sessions.

### Statistics

The x-y coordinate data for one NC subject was not able to be used due to a contaminated digital video, but the object interaction data for this subject were retained. Data were first examined for normality and homogeneity of variance. Two outliers (values greater than 3 standard deviations away from the mean for BD or NC) were identified; the values of these outliers were truncated to 3 standard deviations from the mean, in order to retain their extreme value while preserving power. Mixed analyses of variance (ANOVA) were used, with session as the repeated measure and group (BD vs. NC) as the between-subjects measure. Changes in YMRS scores were analyzed with a repeated-measures ANOVA in BD patients only. Planned pairwise comparisons were Bonferroni-corrected for multiple comparisons. To assess test-retest reliability, Pearson r correlation coefficients were calculated between sessions for the NC subjects. Significance levels were set at p≤.05. All analyses were conducted with SPSS.

## Results

NC and BD subjects were not significantly different in age [t(31) = 1.7, NS] or years of education [t(31) = 1.3, NS]. There was a significant main effect of session on total YMRS scores [F(2, 22) = 4.0, p = .03); Bonferroni pairwise comparisons revealed a non-significant trend of decreasing scores from Session 1 to Session 3 (p = .09). The decrease between Session 1 and 2 and the decrease between Session 2 and 3 were not significant. Mean YMRS scores were as follows: Session 1, 27.2 (SD = 8.2); Session 2, 21.4 (SD = 11.5); and Session 3, 18.7 (SD = 11.2).

**Figure 2:**
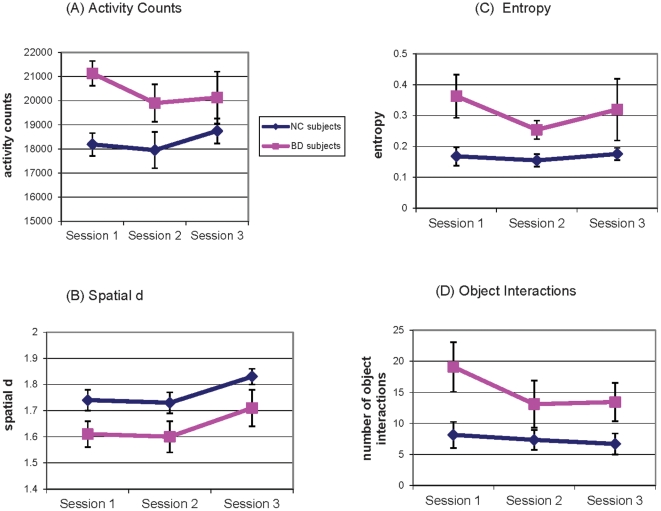
hBPM measures of activity and exploration for manic BD and NC subjects across three sessions. Panel A illustrates that activity counts are higher in manic BD subjects in all sessions. Panel B illustrates that manic BD subjects have lower spatial *d*, reflecting a combination of localized and straight movements, compared to NC subjects who tend to have more localized movements (*d* approaching 2). Spatial *d* for all subjects increases in Session 3, suggesting that all subjects tend to engage in more localized exploration during this third exposure to the hBPM. Panel C illustrates that BD subjects have more unpredictable movements in the hBPM, signified by higher entropy values, than NC subjects. Panel D illustrates that object interactions are significantly higher in manic BD subjects in all three sessions.

Analysis of the hBPM measures indicated a main effect of group on total motor activity [F(1,30) = 8.6, p = .002], such that BD patients showed significantly more activity counts than NC subjects across sessions (Figure 2a), but no main effect of session [F(2,60) = .78, NS] nor a session-by-group interaction [F(2,60) = .87, NS] for counts. With respect to spatial *d*, there was a main effect of session [F(2,60) = 5.9, p = .005] and a main effect of group [F(1,30) = 5.5, p = .03], but no session-by-group interaction [F(2,60) = .02, NS]. BD patients showed lower spatial *d*, signifying a combination of long, straight movements and localized activity throughout all three sessions. In contrast, NC subjects primarily engaged in more circumscribed movements as represented by higher values of spatial *d*. Both groups showed relative increases in spatial *d* (tendency towards smaller, localized movements) during subsequent sessions, especially from Session 2 to Session 3 (pairwise comparison p = .006; Figure 2b). With respect to entropy, there was no main effect of session [F(2,60) = 1.5, NS] nor a session-by-group interaction [F(2,60) = .87, NS]. There was a significant main effect of group on entropy [F(1,30) = 9.3, p = .005], such that BD patients demonstrated higher entropy, signifying more disordered and unpredictable movement in the hBPM relative to NC subjects during all three sessions (Figure 2c).

There was a main effect of group on object interactions [F(1, 31) = 7.0, p = .01] in the absence of a significant main effect of session [F(2,62) = 2.4, p = .10] or a session-by-group interaction [F(2,62) = 1.1, NS]. BD patients showed more object interactions than NC subjects in all three sessions (Figure 2d). Table 2 illustrates positive correlations in NC subjects among the three sessions for the hBPM measures.

**Table 2:** Pearson r correlation coefficients for hBPM measures between sessions for NC subjects (n = 21).

	Session 1/Session 2	Session 2/Session 3	Session 1/Session 3
Counts	.46*	.40	.55*
Spatial *d*	.54*	.52*	.30
Entropy *h*	.39	.56**	.71**
Object Interactions	.73**	.81**	.74**

*p<.05.

**p≤.01.

**Figure 3:**
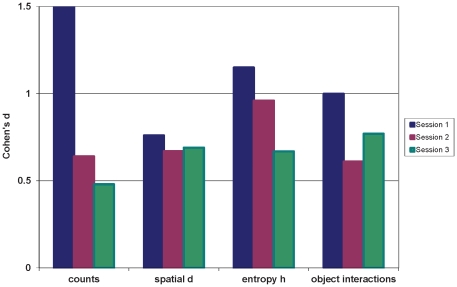
Cohen's d effect sizes for differences between manic BD and NC subjects in hBPM measures across three sessions.

Cohen's d effect sizes were calculated for the differences between BD and NC subjects for the hBPM measures, for all three sessions. Figure 3 illustrates that the magnitude of difference between these two groups was largest in Session 1, but an effect size of at least .50 (medium effect size) was still observed in Sessions 2 and 3.

**Figure 4:**
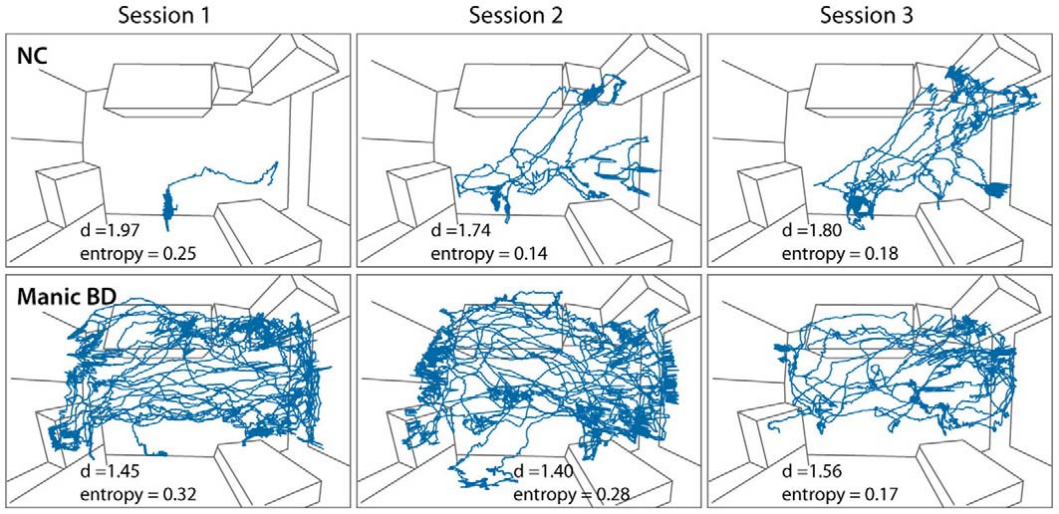
Exploratory activity of one NC subject and one manic BD subject over three sessions in the hBPM. The manic BD subject shows more exploration than the NC subject overall, and the NC subject does not show marked decrease of exploration across the three sessions. Spatial *d* values reflect that the manic BD subject shows localized movements as well as straight movements that cross the hBPM (*d* approaching 1.5) while the majority of the NC subject's activity tends to be more localized and long crossings of the hBPM are absent (*d* approaching 2.0). Entropy values are higher in the manic BD subject for the first two sessions compared to NC subjects and compared to the third session, reflecting relatively more unpredictable movements in the manic BD subject that become more organized and predictable by the third session.

The exploratory patterns of one representative manic BD subject and one representative NC subject are displayed in Figure 4. The manic BD subject shows more exploration than the NC subject, and spatial *d* values approximating 1.5 reflect a combination of straight, long movements that traverse the hBPM as well as localized, small movements to explore objects. Entropy values that are higher than those of the NC subject in the first two sessions reflect a relatively more unpredictable pattern of exploration which becomes more organized and predictable by the third session. The NC subject explores less than the manic BD subject overall but does not demonstrate significantly reduced exploration as the sessions progress. Spatial *d* values closer to 2.0 for the NC subject suggest that localized activity is more prominent and straight crossings of the hBPM are relatively few.

## Discussion

Hyper-exploration, novelty seeking, and increased motor activity are thought to be hallmark features of BD mania [20], [21], [22]. Historically, these characteristics have not been quantified objectively and reliably, hence our rationale for the development of the hBPM [23]. Further, it remains largely unknown to what extent this manic phenotype is altered or reduced with treatment. Additionally, while BD patients exhibit a marked pattern of hyper-exploration in the hBPM, one concern with repeated exposure to this paradigm is that the environment may not retain its novelty across multiple sessions, even with different sets of novel stimuli in the room. Thus our primary aim was to determine if BD patients' activity in the hBPM would remain elevated despite potential changes in the symptoms of treated patients. This study also enabled us to ascertain whether the hBPM is a sensitive and reliable method of quantifying exploratory activity in healthy subjects during repeated testing conditions. Our results support several main conclusions. First, increased activity and exploration of novel stimuli appear to persist in BD patients despite modest initiation of recovery from the manic episode, suggesting that these features of the illness are not immediately responsive to treatment even as manic symptoms begin to abate. The contour maps in Figure 1 illustrate that NC subjects consistently exhibited moderately low exploratory activity in the hBPM, while BD subjects had many entries into sectors that were adjacent to novel objects during all three sessions. It is important to note that YMRS scores for BD patients remained high even during the final test session; per convention YMRS scores of 20 and above are reflective of moderate mania [24] thus a mean score of 18 at Session 3 would suggest a continued hypomanic state. Increased novelty seeking has been observed in “recovered” BD patients [12] as well as in BD patients in the depressed phase [25], suggesting that it may be an enduring feature of BD. Our ongoing hBPM studies on stable euthymic BD patients should shed further light on the state versus trait nature of increased exploration and novelty seeking in this disease.

Although the objects in the hBPM are changed for each of the three sessions in an effort to minimize habituation, subjects are exposed repeatedly to the same physical space with the same expectations (e.g., wait in the room for the experimenter). Thus it was important to examine the impact of repeated exposure to the hBPM. For most of the hBPM-derived measures of exploration and activity, there did not appear to be a prominent dampening or habituation effect of the hBPM environment, at least over three testing sessions. This point is best illustrated by the fact that the healthy subjects did not show dramatic decreases in measures of exploration over repeated testing. The test-retest reliability of the hBPM is also supported by evidence that its measures were generally correlated between sessions in the healthy subjects. Inter-session correlations were weaker in the manic BD subjects (data not shown), which is likely partially attributable to the small sample size of this group. In addition, the decrease in group difference effect sizes across sessions (illustrated in Figure 3) suggests that while manic BD subjects did show modest reductions in exploration and novelty seeking, they remained well above the level of healthy subjects.

Exploration and novelty seeking are putative functions of the brain's reward system, given that the discovery of a new stimulus reinforces further exploration [26]. Dopamine is thought to play a vital role in reinforcement and reward [27]. Neuroimaging [28], [29] and genetic [30] studies implicate dopamine pathways in novelty seeking and the related constructs of exploration and over-activity. Given that the majority of the BD subjects were treated consistently with antipsychotic medications over the three-week course of this study, our results suggest that acute pharmacological blockade of dopamine does not dramatically decrease exploration to the level of healthy volunteers. Considering the literature suggesting that dopamine-related genes such as some polymorphisms of the dopamine transporter gene are uniquely expressed in BD [31], altered dopamine tone may be an important neurobiological feature of the illness of BD and not unique to manic episodes Furthermore, our findings suggest that acute administration of medications in animals may not be the most appropriate way to validate animal models of mania. For example, acute [32], [33], [34], [35] but not chronic [36], [37] lithium reversed stimulant-induced hyperactivity. Given that hyperactivity in manic BD humans is not significantly reversed with acute treatment, these existing animal models of BD require further refinement and validation [38].

With respect to the test-retest stability of the hBPM, the one measure that did reflect a systematic change across time in both BD subjects and healthy volunteers was spatial *d*, which reflects the individual's pattern of exploration. As we reported previously [7] and confirmed here, the exploratory behavior of manic BD patients includes long, straight movements in the hBPM (lower spatial *d*), suggestive of a more expansive approach to exploring the environment than NC subjects, and also smaller localized movements, e.g., to interact with specific objects. Repeated testing in the hBPM reveals that BD patients and healthy comparison subjects shift towards utilization of smaller, more localized movements (higher spatial *d*), most notably between the second and third exposure. This phenomenon is not dissimilar to the typical characteristics of an animal's exploration of a novel field over time: the organism's early movements may consist of straight, directed forays to familiarize itself with the general features of the environment and then return to its home base [39]. In the case of the hBPM, as individuals' familiarity with the novel field increases, their strategy may evolve to smaller movements, potentially reflecting more localized exploration of potentially salient details [7].

This study's primary limitation is the small size of the BD sample. Further, as this was a naturalistic study and BD patients were on a variety of psychotropic medications, the effects of specific agents on measures of activity and exploration are difficult to discern. For example, we cannot draw conclusions about the potential differential impact of mood stabilizing medications alone versus treatment with a combination of a mood stabilizer and an antipsychotic agent. In general we have not observed prominent medication effects on hBPM measures of exploration [7], [10], [11]. Future work might examine whether specific compounds, e.g., valproate versus lithium, differentially affect activity and exploration, thus the anti-manic properties of commonly used medications for BD can be quantifiably compared. The use of the hBPM in controlled clinical trials, e.g., placebo versus active drug, could more sensitively test whether increased exploration and novelty seeking change with drug treatment. In a related point, YMRS scores did not decrease to euthymic levels in this sample of manic subjects, limiting our ability to assess the relationship between exploration and changes in mood state. Future studies with a longer follow-up period and/or direct comparison of manic versus euthymic and even depressed BD individuals are warranted; the latter investigations are ongoing in our laboratory. Finally, the relatively low baseline levels of exploration in the non-patient comparison subjects may have contributed to the putative lack of habituation to the hBPM environment in this group.

In conclusion, this report provides an initial validation of the utility of the hBPM in a repeated-measures design and illustrates that the increased exploration and novelty seeking characteristic of BD mania are not rapidly ameliorated with treatment. The hBPM represents a valuable tool for both cross-species research and clinical studies, as an adaptation of animal open field paradigms that may be applied to assess exploration and novelty seeking in multiple neuropsychiatric disorders.
